# Heterogeneity of depressive symptoms and associated factors in Korean adults: a latent class analysis using a national data survey

**DOI:** 10.7586/jkbns.26.007

**Published:** 2026-05-27

**Authors:** Hohyun Seong

**Affiliations:** College of Nursing, Chonnam National University, Gwangju, Korea

**Keywords:** Depression, Latent class analysis, Life style, Obesity, Sleep

## Abstract

**Purpose:**

This study aimed to identify latent classes of depressive symptoms among Korean adults and to examine the sociodemographic and biobehavioral factors associated with each subgroup.

**Methods:**

This secondary analysis used data from 4,646 adults who participated in the 2022 Korea National Health and Nutrition Examination Survey. Latent class analysis was applied to the 9 items of the Patient Health Questionnaire-9 to identify distinct subgroups. Multinomial logistic regression was then performed to determine the factors associated with class membership.

**Results:**

Three distinct latent classes were identified: the low-risk group (61.1%), the somatic symptom group (31.0%), and the global depression group (7.9%). The somatic symptom group, which comprised the largest proportion of symptomatic individuals, was characterized predominantly by physical complaints. In contrast, the global depression group showed high probabilities across all symptom domains. Female sex and shorter sleep duration emerged as shared risk factors for both depressive classes. However, unhealthy lifestyle behaviors, including high-risk drinking and obesity, were associated exclusively with the global depression group, compared with the low-risk group, and were not associated with the somatic symptom group.

**Conclusion:**

These findings confirm the heterogeneity of depressive symptoms among Korean adults. Notably, unhealthy lifestyle behaviors such as high-risk drinking and obesity were associated with the most severe global manifestation of depression rather than with the somatic subtype. These results support the need for stratified interventions. Although sleep hygiene education may serve as a universal preventive strategy, targeted lifestyle modifications may be particularly important for individuals in the high-risk global depression group.

## INTRODUCTION

Depression is a leading cause of disability worldwide and a critical public health concern in South Korea. Over the past decade, the prevalence of depressive symptoms in Korea has typically shown an upward trend, emerging as a major contributor to reduced quality of life and the nation's alarmingly high suicide rate [1,2]. In clinical and research settings, depressive symptoms are commonly assessed using screening tools such as the Patient Health Questionnaire-9 (PHQ-9). Traditionally, these assessments rely on a variable-centered approach, summing item scores to yield a total severity index [3]. While effective for gauging the overall intensity of distress, this cumulative approach often masks the substantial heterogeneity of depression. Two individuals with the identical total score may present with entirely different symptom profiles [4,5]—one dominated by affective symptoms like sadness and anhedonia, and the other by somatic complaints such as sleep disturbances, fatigue, and appetite changes.

This heterogeneity is particularly relevant in the Asian cultural context. In South Korea, where Confucian values emphasize emotional restraint, mental health stigma remains a barrier to help-seeking. Consequently, psychological distress is frequently expressed through physical symptoms rather than explicit verbalizations of mood, a phenomenon often described as ‘somatization’ or ‘masked depression’ [6]. Recognizing these distinct patterns is crucial because the underlying risk factors and optimal treatment responses may differ significantly depending on whether depression manifests primarily through somatic dysregulation or affective disturbance.

Depression is a multifactorial disorder shaped by a complex interplay of sociodemographic characteristics, modifiable lifestyle behaviors, and physiological health status. Sociodemographic factors are not merely control variables but are hypothesized to influence symptom presentation. For instance, older adults may predominantly exhibit somatic complaints due to age-related physiological changes or the stigma surrounding mental health [7]. Furthermore, socioeconomic status is a critical factor. Financial deprivation and chronic life stress may predispose individuals to somatic manifestations of distress [8]. Gender-specific socialization also plays a pivotal role, influencing not only the higher prevalence observed in women [9-11] but also the mode of symptom expression by shaping whether distress is internalized somatically or articulated affectively [12]. Building upon these sociodemographic characteristics, bio-behavioral factors such as high-risk drinking, obesity, and sleep duration are increasingly recognized as key correlates of depressive phenotypes. High-risk drinking, often utilized as a maladaptive coping mechanism, may aggravate affective instability through neurochemical disruption [13-15]. In contrast, obesity is linked to the 'immuno-metabolic' hypothesis, where chronic low-grade inflammation triggers 'sickness behavior'—characterized by physical symptoms such as fatigue and lethargy—thereby predisposing individuals to somatic-dominant profiles [16,17]. Sleep disturbances also act as a bidirectional risk factor, disrupting circadian rhythms and precipitating both somatic and affective manifestations [18-20]. Additionally, nutritional biomarkers like vitamin D have drawn attention for their potential neuroprotective roles, although epidemiological evidence remains inconsistent [21,22]. Since these physiological mechanisms can result in different symptom patterns, relying solely on total severity scores may mask the underlying heterogeneity. This highlights the need for person-centered approaches like Latent Class Analysis (LCA) to identify specific subgroups characterized by these unique bio-behavioral and sociodemographic profiles.

To disentangle these complex relationships, a person-centered methodological approach is imperative. While variable-centered methods provide a useful summary of severity, they often obscure the qualitative heterogeneity of depression—specifically, the distinct symptom profiles that may require different intervention strategies. LCA addresses this limitation by identifying unobserved subgroups (latent classes) based on unique symptom patterns [23]. Although previous studies have applied LCA to depression [24,25], they primarily focused on specific clinical samples or limited symptom domains. A critical gap remains in the literature, as few studies have utilized recent, nationally representative data to systematically examine how comprehensive sociodemographic characteristics and bio-behavioral factors are differentially associated with specific depressive subtypes. Addressing this gap is essential for establishing an empirical basis for precision public health interventions in the general population.

Therefore, this study aimed to (1) identify distinct latent classes of depressive symptoms among Korean adults; (2) compare the sociodemographic and bio-behavioral characteristics of these classes; and (3) examine the specific associations of lifestyle behaviors—including high-risk drinking, obesity, sleep duration, and vitamin D status—with each identified latent class. By elucidating the unique risk architectures associated with these specific latent phenotypes, this study aims to provide the empirical basis for developing targeted, precision-based public health interventions tailored to the diverse manifestations of depression in the Korean population.

## METHODS

### 1. Study design

This study employed a descriptive, cross-sectional design involving a secondary analysis of data from the 2022 Korea National Health and Nutrition Examination Survey (KNHANES). The KNHANES is a nationwide surveillance system conducted annually by the Korea Disease Control and Prevention Agency (KDCA) to examine the health and nutritional status of the South Korean population [26]. The survey comprises three comprehensive components: a health interview, a health examination, and a nutrition survey.

### 2. Setting and samples

The KNHANES utilizes a stratified, multistage clustered probability sampling design based on data from the National Census to ensure a nationally representative sample of non-institutionalized civilians. For the current analysis, we utilized the 2022 dataset, which is publicly available. Initially, the 2022 KNHANES included a total of 6,265 participants. From this pool, we excluded 943 individuals aged under 19 years, resulting in 5,322 eligible adult participants. Subsequently, 676 participants were excluded due to missing data on the PHQ-9 or other key health-related variables. Consequently, the final analytical sample comprised 4,646 adults aged 19-80 years.

### 3. Measurements

#### 1) Sociodemographic characteristics

Sociodemographic variables included age, sex, education, marital status, income, and employment status. Age was dichotomized into adults (19–64 years) and older adults (≥ 65 years). Education level was reclassified into four categories (≤ elementary school, middle school, high school, and ≥ college or university) from the original eight categories since the proportion of those with less than elementary school and graduate school was very low (2.9% and 5.0%, respectively). Marital status was grouped into three categories: married or cohabiting, previously married (widowed, divorced, or separated), and single. Employment status was classified as 'employed' or 'unemployed' based on engagement in paid work for at least one hour in the previous week. Household income was standardized by age and sex to classify participants into four quartile-based groups: lowest relative income, lower-middle relative income, upper-middle relative income, and highest relative income.

#### 2) Bio-behavioral variables

Bio-behavioral characteristics included smoking, alcohol consumption, physical activity, obesity, sleep duration, and vitamin D status. Smoking was dichotomized into current smokers versus non-smokers (including former and never smokers). High-risk drinking was defined as consuming seven or more standard drinks for men and five or more for women on at least two occasions per week [27]. Physical activity was assessed using the 9-item International Physical Activity Questionnaire across work, leisure, and transportation domains, with vigorous activity minutes weighted by a factor of two to calculate moderate-intensity equivalents. Based on this total volume, participants were dichotomized according to World Health Organization (WHO) guidelines into those meeting the recommendation (> 150 min/week) and those who did not (≤ 150 min/week) [28].

For obesity, we applied the WHO Asia-Pacific perspective, classifying participants with a body mass index (BMI) of ≥ 25 kg/m² as obese [29]. Sleep duration was assessed based on participants' self-reported usual bedtime and wake-up times for both weekdays (working days) and weekends (non-working days), rather than simple estimates of total sleep hours. We calculated the daily sleep duration from these reported times and derived the average daily sleep duration using a weighted formula to account for weekly variations: [(weekday sleep duration × 5) + (weekend sleep duration × 2)] / 7 [30]. In the KNHANES data, liquid chromatography-tandem mass spectrometry was employed to quantify serum 25-hydroxyvitamin D [25(OH)D] levels, with the final concentration determined by summing 25(OH)D2 and 25(OH)D3. Based on these levels, vitamin D status was categorized into three groups: normal (≥ 30 ng/m), insufficiency (20–29 ng/mL), and deficiency (< 20 ng/mL) [22].

#### 3) Depressive symptoms

Depressive symptoms were measured using the PHQ-9 [3]. Participants rated the frequency of their symptoms during the past 2 weeks using a 4-point Likert scale (0–3), resulting in a total score between 0 and 27. The Cronbach’s alpha coefficient for the PHQ-9 in this study was .80, indicating good reliability.

### 4. Data collection

Data from the 2022 KNHANES were collected through health interviews, health examinations, and nutrition surveys conducted by trained medical staff and interviewers. The health interview and nutrition survey were performed via face-to-face interviews and self-administered questionnaires, while the health examination included physical measurements and blood sampling.

### 5. Ethical considerations

Approval for the study protocol was obtained from the Chonnam National University Institutional Review Board (IRB No. 1040198-251202-HR-241-0). The raw data from the KNHANES IX-1 (2022) were provided by the KDCA following approval under their data disclosure protocols. To ensure privacy, the dataset had been subjected to de-identification measures, such as top- and bottom-coding and recategorization, before being released to researchers.

### 6. Data analysis

Data analysis was executed utilizing Stata version 17.0 (StataCorp, College Station, TX, USA) and Mplus version 8.0 (Muthén & Muthén, Los Angeles, CA, USA). All analyses applied sample weights to account for the complex sampling design of the KNHANES, ensuring representative estimates and robust standard errors.

First, complex sample descriptive statistics were computed to summarize the sociodemographic characteristics, health behaviors, and clinical profiles of the study population. Second, LCA was performed using Mplus to identify distinct subgroups of individuals sharing similar patterns of depressive symptoms. The nine items of the PHQ-9 were recoded into binary variables (0 = absent, 1 = present) to serve as categorical indicators for the LCA model. We estimated models with one to five classes to determine the optimal number of latent phenotypes. Model selection was guided by a combination of statistical fit indices and substantive interpretability. Model fit was evaluated by minimizing values across several indicators, including the Akaike Information Criterion (AIC), Bayesian Information Criterion (BIC), and sample-size adjusted BIC (saBIC). Additionally, the bootstrapped likelihood ratio test and the Vuong-Lo-Mendell-Rubin likelihood ratio test were used to assess whether a model with k classes provided a significantly better fit than a model with k-1 classes (*p* < .050) [31]. Classification quality was evaluated using entropy, with values closer to 1 indicating clear separation between classes. Considering both statistical criteria and clinical interpretability, a 3-class model was selected as the optimal solution.

Finally, multinomial logistic regression analysis was performed using Stata to examine the associations between the identified latent classes (dependent variable) and covariates. Statistical significance was set at *p* < .050.

## RESULTS

### 1. General characteristics of the participants

The general characteristics of the 4,646 participants are presented in Table 1. The mean age of the participants was 48.40 ± 0.47 years, with the majority (80.8%) falling into the 19–64 age group. The proportion of men (50.1%) was slightly higher than that of women (49.9%). Regarding socioeconomic factors, the largest proportion of respondents were married or living with a partner (62.9%), employed (65.2%), and had a college degree or higher (46.9%). For household income, the high-income group accounted for the largest share at 32.7%.

In terms of bio-behavioral characteristics, the prevalence of current smoking and high-risk drinking was 17.1% and 17.8%, respectively. Approximately 37.3% of the participants were classified as obese based on a BMI ≥ 25 kg/m². The mean sleep duration was 6.99 ± 0.02 hours per day. Notably, regarding vitamin D status, only 21.9% of participants had normal levels, while the prevalence of vitamin D deficiency and insufficiency was 45.7% and 32.4%, respectively. The mean PHQ-9 score for the total sample was 2.42 ± 0.06.

### 2. Latent classes of depressive symptoms

Table 2 summarizes the model fit indices for one- to five-class models estimated to determine the optimal number of latent phenotypes. While the AIC, BIC, and saBIC values decreased consistently as the number of classes increased, the LMR-LRT indicated that the 3-class model provided a significantly better fit than the 2-class model (*p* = .007). In contrast, the 4-class model did not show a statistically significant improvement over the 3-class model (*p* = .136). The entropy for the 3-class model was 0.81, indicating high classification accuracy. Based on these statistical indices and clinical interpretability, the 3-class solution was selected.

**Figure 1.**
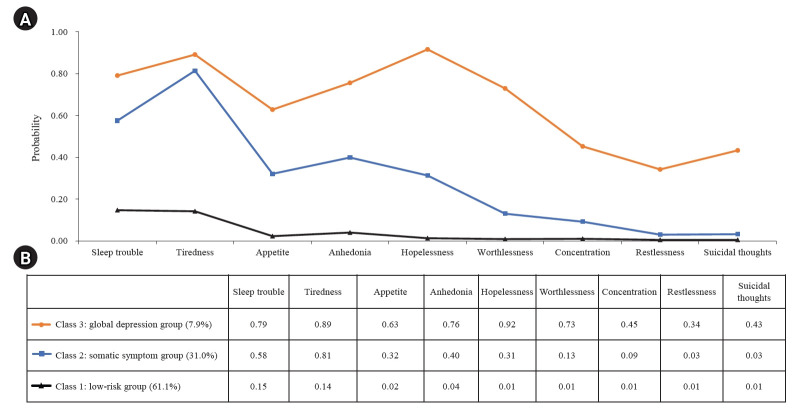
Item-response probabilities for the three latent classes of depressive symptoms. (A) Line graph of response probabilities for the nine PHQ-9 items. (B) Detailed probabilities for each class. The items on the x-axis are grouped into two domains to highlight symptom patterns: somatic symptoms (sleep trouble, tiredness, and appetite) and affective/cognitive symptoms (anhedonia, hopelessness, worthlessness, concentration, restlessness, and suicidal thoughts).

Figure 1 illustrates the item-response probabilities for the identified classes. Class 1 (61.1%) was labeled the ‘low-risk group,’ as individuals in this class had near-zero probabilities of endorsing any depressive symptoms. Class 2 (31.0%) was labeled the ‘somatic symptom group.’ Individuals in this group were distinguished by high probabilities of somatic complaints—such as sleep trouble, tiredness, and appetite changes—while reporting relatively low probabilities for core affective symptoms like anhedonia and depressed mood. Class 3 (7.9%) was labeled the ‘global depression group.’ This group exhibited the most severe profile with high probabilities across all nine items, encompassing both somatic and affective domains.

### 3. Comparison of characteristics among latent classes

Sociodemographic and bio-behavioral characteristics differed significantly across the three latent classes (Table 3). Significant differences were observed in sex, age, marital status, education, income, employment status, high-risk drinking, sleep duration, and vitamin D status (all *p* < .050). Specifically, the somatic symptom group (Class 2) was predominantly composed of women (63.5%), whereas the low-risk group had a relatively balanced gender distribution (women: 51.3%). The global depression group (Class 3) also exhibited the highest proportion of women (69.1%) among the three classes. Notably, high-risk drinking was most prevalent in the global depression group (17.5%), followed by the low-risk group (11.8%) and the somatic symptom group (10.9%) (*χ*^2^ =18.65, *p* = .003). Sleep duration was significantly shorter in Class 2 (6.89 ± 0.03 hours) and Class 3 (6.80 ± 0.08 hours) compared to Class 1 (7.06 ± 0.02 hours; *p* < .001). Although the distribution of Vitamin D status differed statistically (*p* = .039), the prevalence of deficiency was higher in both the somatic symptom group (43.3%) and the global depression group (43.4%) compared to the low-risk group (38.9%).

### 4. Factors associated with latent classes

As shown in Table 4, multinomial logistic regression was conducted to determine factors for each latent class relative to the low-risk group (Class 1). The overall fit of the multinomial logistic regression model was assessed using the design-adjusted Wald F-test, taking into account the complex sampling design. The model was found to be statistically significant (*F* (40, 126) = 7.56, *p* < .001), indicating that the selected sociodemographic and bio-behavioral factors are significantly associated with latent class membership. Specifically, women and shorter sleep duration emerged as shared risk factors for both depressive classes. Compared to men, women had higher odds of belonging to both the somatic symptom group (OR = 1.99, 95% CI: 1.62–2.46) and the global depression group (OR = 2.88, 95% CI: 1.94–4.26). In addition, age was a significant sociodemographic factor. Compared to adults (19–64 years), older adults (≥ 65 years) had significantly lower odds of belonging to both the somatic symptom group (OR = 0.51, 95% CI: 0.38–0.68) and the global depression group (OR = 0.32, 95% CI: 0.18–0.59). Marital status also emerged as a significant sociodemographic factor. Compared to single participants, those who were married or living with a partner had significantly lower odds of belonging to the somatic symptom group (OR = 0.57, 95% CI: 0.46–0.72) and the global depression group (OR = 0.35, 95% CI: 0.23–0.55). Individuals who were widowed, divorced, or separated also exhibited lower odds for the somatic symptom group (OR = 0.65, 95% CI: 0.46–0.92), but this association was not significant for the global depression group. Longer sleep duration was associated with a significantly lower likelihood of membership in both the somatic symptom group (OR = 0.83, 95% CI: 0.77–0.90) and the global depression group (OR = 0.75, 95% CI: 0.65–0.87).

However, distinct bio-behavioral and socioeconomic risk profiles were observed for the most severe subtype. High-risk drinking (OR = 1.91, 95% CI: 1.30–2.79) and obesity (OR = 1.49, 95% CI: 1.10–2.03) were identified as significant factors specific to the global depression group. Additionally, socioeconomic indicators were uniquely associated with this group; employment (OR = 0.72, 95% CI: 0.53–0.98) and high household income (OR = 0.55, 95% CI: 0.31–0.96) were significant protective factors against membership in the global depression group. In contrast, these factors were not significantly associated with the somatic symptom group (high-risk drinking: OR = 0.94; obesity: OR = 1.19; employment: OR = 1.18; high income: OR = 0.73). Regarding physiological indicators, although vitamin D status showed significant differences based on univariate findings, the adjusted model revealed no significant relationship with any latent class.

## DISCUSSION

This study utilized LCA to identify distinct phenotypes of depressive symptoms among Korean adults using data from the 2022 KNHANES. By moving beyond a variable-centered approach that relies solely on cumulative symptom scores, this study elucidates the heterogeneity of depression in the general population. We identified three distinct latent classes: a ‘low-risk group’ (61.1%), a ‘somatic symptom group’ (31.0%), and a ‘global depression group’ (7.9%). These findings highlight that depressive symptoms do not manifest uniformly across individuals; rather, they cluster into varying profiles with unique sociodemographic and bio-behavioral correlates.

The characterization of the somatic symptom group (Class 2), which accounted for nearly one-third of the population, is particularly noteworthy. Unlike the global depression group, which exhibited high probabilities across all symptom domains, the somatic symptom group displayed a discordant pattern. Individuals in this group endorsed high levels of physical symptoms, such as sleep trouble, tiredness, and appetite changes, yet reported relatively low levels of core affective symptoms like anhedonia and depressed mood [24]. This pattern aligns with the concept of ‘masked depression’ or somatization documented in previous studies, often observed in East Asian populations [5,32]. Specifically, in the Korean sociocultural context, traditional norms often emphasize emotional restraint, which contributes to a persistent stigma against mental illness [33,34]. Due to these factors, individuals may suppress affective symptoms (e.g., depressed mood) and instead manifest their psychological distress primarily through physical complaints [5,6]. Consequently, a significant proportion of depressive patients might be misidentified in primary care settings if clinicians focus exclusively on affective complaints. These findings suggest that the somatic symptom group likely represents a distinct depressive phenotype rooted in cultural expressions of distress, rather than non-pathological fatigue. Therefore, nursing assessments must extend beyond standard mood inquiries. Nurses should adopt a culturally sensitive approach by proactively assessing for somatic manifestations of depression [35]— such as unexplained chronic pain, gastrointestinal distress, or lethargy—especially in women and older adults who may be reluctant to express emotions due to stigma. Regarding interventions, therapeutic strategies for this group should prioritize validating their physical distress rather than attributing it solely to psychological causes. Integrated nursing interventions that combine sleep hygiene education with somatic-focused stress management—such as progressive muscle relaxation or mindfulness-based stress reduction—may be more effective than standard preventive strategies alone [36,37].

Regarding sociodemographic predictors, distinct risk profiles emerged for each class. Consistent with extensive epidemiological evidence, women were a robust predictor for membership in both depressive classes [10]. This gender disparity may be attributed to a combination of biological vulnerabilities and sociocultural stressors, such as the dual burden of domestic and professional labor, which disproportionately affects women in South Korea [9]. Interestingly, adults (19–64 years) had a higher likelihood of membership in the global depression group compared to the low-risk group. This finding resonates with recent reports highlighting the increasing mental health burden on young and middle-aged adults who face intensifying socioeconomic challenges, including housing instability, competitive job markets, and financial insecurity [38]. These structural stressors likely contribute to the comprehensive syndrome of emotional and physical depletion observed in the global depression group. In contrast, older age appeared to be a protective factor against this global subtype, potentially reflecting the ‘happiness curve’ or greater emotional regulation strategies developed in later life, although the protective effect was less pronounced for somatic manifestations [39].

Beyond age and gender, our findings highlight the critical role of social and economic stability in shaping depressive phenotypes. Being married or living with a partner emerged as a universal protective factor, significantly lowering the odds of membership in both the somatic symptom and global depression groups. This suggests that spousal support serves as a fundamental emotional buffer that mitigates psychological distress regardless of its manifestation [40]. In contrast, socioeconomic markers—specifically employment and high household income—were uniquely associated with reduced risk for the global depression group. This distinction highlights the socioeconomic vulnerability of the most severe subtype [41]; financial deprivation and unemployment likely act as chronic stressors that compound bio-behavioral risks, driving the progression of depression into its most pervasive and severe form.

A novel and critical finding of this study is the specific association between bio-behavioral factors—high-risk drinking and obesity—and the most severe phenotype, the global depression group (Class 3). While sociodemographic factors broadly predicted depression risk, these bio-behavioral factors were uniquely associated with the global depression group, distinguishing it from the somatic symptom group. This suggests that unhealthy lifestyle behaviors are not only linked to somatic symptoms but are associated with the transition of depression into its most severe and pervasive form.

The strong link between high-risk drinking and global depression suggests a complex reciprocal relationship. Alcohol is frequently used as a maladaptive coping mechanism, or ‘self-medication,’ to alleviate stress. However, excessive alcohol consumption disrupts neurochemical balance and triggers systemic inflammation, which can exacerbate both somatic and affective symptoms, potentially leading to a more severe, global presentation [13,14]. Similarly, the specific association with obesity in the global depression group supports the ‘immuno-metabolic’ hypothesis of depression. Adipose tissue secretes pro-inflammatory cytokines that can induce ‘sickness behavior’ and alter mood regulation [16]. Our findings imply that metabolic dysregulation and inflammation associated with obesity may be closely linked to a global depressive subtype characterized by both intense physical and emotional distress. Therefore, for this high-risk group, interventions targeting metabolic health and alcohol reduction are essential alongside psychiatric treatment.

Sleep duration emerged as a fundamental transdiagnostic factor across all groups. Shorter sleep duration was strongly associated with increased odds of membership in both the somatic symptom and global depression groups. This finding reinforces the well-documented bidirectional relationship between sleep and mental health [18,19]. The universality of this risk factor suggests that promoting adequate sleep hygiene should be a core component of public health strategies for depression prevention, regardless of the specific symptom subtype. In terms of vitamin D, although deficiency was more prevalent in the depressive groups in univariate analyses, this association was attenuated in the adjusted multinomial models. This finding reflects the inconsistent epidemiological evidence regarding vitamin D’s independent role in depression [21,42]. Our results suggest that the relationship between vitamin D and depressive phenotypes is likely confounded by other bio-behavioral factors, such as physical activity or outdoor exposure, rather than acting as a direct independent correlate. In this study, vitamin D status may serve more as a proxy for a generally healthy lifestyle rather than a specific etiological factor for distinct depressive subtypes.

This study has several limitations. First, due to the cross-sectional design, causal relationships between bio-behavioral variables and depressive phenotypes cannot be established. Specifically, it remains unclear whether high-risk drinking and obesity are antecedents to the severe global depressive phenotype or consequences of the condition, necessitating longitudinal research to clarify directionality. Second, depressive symptoms were assessed using the PHQ-9, a self-report screening instrument, rather than a structured diagnostic interview. While the PHQ-9 is a validated tool, it may not capture the full clinical complexity of depressive disorders compared to clinician-administered evaluations. Third, although we adjusted for a wide range of covariates, potential residual confounders—such as psychosocial stress, social support systems, and family history of mental illness—were not available in the dataset and thus could not be included in the analysis. Despite these limitations, our findings confirm the heterogeneity of depression in Korean adults and underscore the need for stratified interventions: targeted bio-behavioral modifications for the high-risk global depression group and universal sleep promotion for all.

## CONCLUSION

This study confirms the heterogeneity of depressive symptoms among Korean adults, identifying three distinct latent classes: a low-risk group, a somatic symptom group, and a global depression group. Our findings reveal that these subgroups are differentiated not only by symptom profiles but also by specific risk factors. Notably, high-risk drinking and obesity were identified as significant factors associated with the global depression group compared to the low-risk group, whereas these associations were not observed for the somatic symptom group. This highlights that unhealthy lifestyle behaviors and metabolic dysregulation are intricately linked to the aggravation of depression into its most severe and pervasive form. Furthermore, sleep duration emerged as a fundamental common determinant. Adequate sleep significantly lowered the probability of membership into both depressive subgroups, highlighting its function as a universal protective factor. These findings advocate for a stratified approach to mental health interventions. Clinically, while practitioners must remain sensitive to the ‘masked’ presentation of the somatic symptom group to ensure early detection, therapeutic strategies for the high-risk global depression group should prioritize bio-behavioral modifications—specifically alcohol reduction and weight management. Simultaneously, public health strategies should promote sleep hygiene education as a foundational preventative measure for the general population, regardless of the depressive subtype.

## Figures and Tables

**Table 1.** General Characteristics of the Participants (N = 4,646)

Characteristics	Categories	n^†^	(%)^‡^
Sociodemographic characteristics			
Sex	Men	2,021	50.1
	Women	2,625	49.9
Age (years, mean ± SE^§^)		48.40 ± 0.47	
	19-64	3,272	80.8
	≥ 65	1,374	19.2
Marital status^‖^	Single	906	25.9
	Married / living with partner	3,083	62.9
	Widowed, divorced, separated	655	11.2
Education^‖^	≤ Elementary school	815	11.2
	Middle school	387	6.7
	High school	1,539	35.2
	≥ College or university	1,904	46.9
Income (relative quartile)^‖^	Lowest	906	15.2
	Lower-middle	1,121	22.1
	Upper-middle	1,264	30.0
	Highest	1,352	32.7
Employment status	Unemployed	1,767	34.8
	Employed	2,879	65.2
Bio-behavioral characteristics			
Smoking^‖^	Former or never smoker	3,939	82.9
	Current smoker	700	17.1
High-risk drinking^‖^	Yes	556	17.8
	No	2,753	82.2
Physical activity^‖^	Yes	2,153	46.4
	No	2,483	53.6
Obesity (based on BMI)	Non-obese (< 25 kg/m^2^)	2,979	62.7
	Obese (≥ 25 kg/m^2^)	1,667	37.3
Sleep duration (hrs/day, mean ± SE^§^)		6.99 ± 0.02	
Vitamin D status	Normal	1,176	21.9
	Deficiency	1,888	45.7
	Insufficiency	1,582	32.4
PHQ-9 score (mean ± SE^§^)		2.42 ± 0.06	

SE = Standard error; BMI = Body mass index; PHQ-9 = 9-item Patient Health Questionnaire.

^†^Unweighted sample size; ^‡^Weighted %; ^§^Estimated mean & SEs; ^‖^The numbers may not sum to the total due to missing data.

**Table 2.** Model Fit Indices for Latent Class Analysis

Number of latent classes	Log-likelihood	AIC	BIC	saBIC	Entropy	LMR-LRT (*p*)	BLRT (*p*)
1	−17418.08	34854.16	34912.15	34883.55	-	-	-
2	−14834.14	29706.28	29828.71	29768.34	0.75	< .001	< .001
3	−14512.96	29083.92	29270.79	29178.64	0.81	.007	< .001
4	−14416.18	28910.36	29161.67	29037.74	0.77	.136	.134
5	−14372.81	28843.63	29159.38	29003.67	0.73	.214	.211

AIC = Akaike information criterion; BIC = Bayesian information criterion; saBIC = Sample-size adjusted BIC; LMR-LRT = Lo-Mendell-Rubin adjusted likelihood ratio test; BLRT = Bootstrapped likelihood ratio test.

**Table 3.** Comparison of Characteristics among Latent Classes (N = 4,646)

Variables	Class 1	Class 2	Class 3	F/ χ^2^	*p*
	(n = 2,837, 61.1%)	(n = 1,443, 31.0%)	(n = 366, 7.9%)		
Sex^†^				55.96	< .001
Men	1,381 (48.7)	527 (36.5)	113 (30.9)		
Women	1,456 (51.3)	916 (63.5)	253 (69.1)		
Age (years)^†^				77.84	< .001
19-64	1,851 (65.2)	1,146 (79.4)	275 (75.1)		
≥ 65	986 (34.8)	297 (20.6)	91 (24.9)		
Marital status^†, ‡^				147.16	< .001
Single	419 (14.8)	385 (26.7)	102 (27.9)		
Married/ living with partner	2,042 (72.0)	864 (59.9)	177 (48.3)		
Widowed, divorced, separated	374 (13.2)	194 (13.4)	87 (23.8)		
Education^†^				29.53	.001
≤ Elementary school	525 (18.5)	210 (14.6)	80 (21.9)		
Middle school	260 (9.2)	99 (6.9)	28 (7.7)		
High school	944 (33.3)	467 (32.3)	128 (35.0)		
≥ College or university	1,107 (39.0)	667 (46.2)	130 (35.4)		
Income (relative quartile)^†^				37.20	< .001
Lowest	544 (19.2)	265 (18.4)	97 (26.5)		
Lower-middle	682 (24.0)	337 (23.4)	102 (27.9)		
Upper-middle	744 (26.2)	429 (29.8)	91 (24.9)		
Highest	866 (30.6)	410 (28.4)	76 (20.7)		
Employment status^†^				14.85	.002
Unemployed	1,086 (38.3)	507 (35.1)	174 (47.5)		
Employed	1,751 (61.7)	936 (64.9)	192 (52.5)		
Smoking^†^				5.94	.157
Former or never smoker	2,418 (85.3)	1,224 (85.0)	297 (81.4)		
Current smoker	416 (14.7)	216 (15.0)	68 (18.6)		
High-risk drinking^†^				18.65	.003
Yes	335 (11.8)	157 (10.9)	64 (17.5)		
No	2,502 (88.2)	1,286 (89.1)	302 (82.5)		
Physical activity^†^				11.99	.019
Yes	1,250 (44.1)	727 (50.5)	176 (48.5)		
No	1,582 (55.9)	714 (49.5)	187 (51.5)		
Obesity^†^				0.75	.776
Non-obese	1,827 (64.4)	917 (63.5)	235 (64.2)		
Obese	1,010 (35.6)	526 (36.5)	131 (35.8)		
Sleep duration (hrs/day, mean ± SE^§^)	7.06 ± 0.02	6.89 ± 0.03	6.80 ± 0.08	8.84	< .001
Vitamin D status^†^				14.03	.039
Normal (≥ 30 ng/m)	751 (26.5)	327 (22.7)	98 (26.8)		
Deficiency (< 20 ng/mL)	1,105 (38.9)	624 (43.3)	159 (43.4)		
Insufficiency (20–29 ng/mL)	981 (34.6)	491 (34.0)	109 (29.8)		

Values are presented as n (%) or mean ± SE. Class 1 = Low-risk group; Class 2 = Somatic symptom group; Class 3 = Global depression group; SE = Standard error.

^†^Unweighted sample size (weighted %); ^‡^The numbers may not sum to the total due to missing data; ^§^Estimated SEs.

**Table 4.** Multinomial Logistic Regression of Factors Associated with Latent Classes

Variables	Somatic symptom group (Class 2)		Global depression group (Class 3)	
	OR	95% CI (Low, High)	OR	95% CI (Low, High)
Sex (ref. = men)	1.99^**^	1.62, 2.46	2.88^**^	1.94, 4.26
Age (ref. = 19-64 years)	0.51^**^	0.38, 0.68	0.32^**^	0.18, 0.59
Marital status (ref. = single)				
Married / living with partner	0.57^**^	0.46, 0.72	0.35^**^	0.23, 0.55
Widowed, divorced, separated	0.65^*^	0.46, 0.92	0.84	0.49, 1.43
Education (ref. = ≤ Elementary school)				
Middle school	1.02	0.61, 1.71	1.46	0.71, 2.97
High school	1.15	0.78, 1.71	1.06	0.60, 1.90
≥ College or university	1.41	0.95, 2.09	1.21	0.66, 2.24
Income (ref. = lowest)				
Lower-middle	0.99	0.71, 1.39	1.07	0.64, 1.77
Upper-middle	0.91	0.68, 1.23	0.66	0.40, 1.08
Highest	0.73	0.53, 1.01	0.55^*^	0.31, 0.96
Employment status (ref. = unemployed)				
Employed	1.18	0.97, 1.44	0.72^*^	0.53, 0.98
Smoking (ref. = former never smoker)				
Current smoker	1.26	0.99, 1.60	1.49	0.88, 2.54
High-risk drinking (ref. = no)				
Yes	0.94	0.72, 1.23	1.91^**^	1.30, 2.79
Obesity (ref. = non-obese)				
Obese	1.19	0.98, 1.45	1.49^*^	1.10, 2.03
Physical activity (ref. = no)				
Yes	1.10	0.91, 1.33	0.99	0.72, 1.37
Vitamin D status (ref. = normal)				
Deficiency	1.24	0.95, 1.62	1.05	0.66, 1.66
Insufficiency	1.27	0.96, 1.67	1.11	0.68, 1.80
Sleep duration	0.83^**^	0.77, 0.90	0.75^**^	0.65, 0.87

Reference class = low-risk group (Class 1). ref. = Reference group; OR = Odds ratio; CI = Confidence interval.

^*^p < .050, ^**^p < .001.
